# The founder sociality hypothesis

**DOI:** 10.1002/ece3.8143

**Published:** 2021-10-16

**Authors:** James Brooks, Shinya Yamamoto

**Affiliations:** ^1^ Wildlife Research Center Kyoto University Kyoto Japan; ^2^ Institute for Advanced Study Kyoto University Kyoto Japan

**Keywords:** bonobos, dogs, extended evolutionary synthesis, intergroup relations, self‐domestication, tolerance

## Abstract

In this review, we propose that the social dynamics of founder populations in novel and newly available environments can have critical effects in shaping species' sociality and can produce long‐lasting changes in social structure and behavior. For founder populations which expand into an underexploited niche separated from the parent population, the necessity of bond formation with strangers, lack of clear territories, and initial abundance of resources can lead to altered initial social dynamics to which subsequent generations adapt. We call this the founder sociality hypothesis. After specifying the theoretical reasoning and mechanism of effect, we focus on three particular cases where the social dynamics of founder populations may have a central role in explaining their modern behavioral ecology. In particular, we develop and review evidence for three predictions of the founder sociality hypothesis in territorial, mixed‐sex group forming species: relatively stronger social bonds in the dispersing sex with relatively weaker bonds in the nondispersing sex, reduced territoriality, and increased social tolerance. We briefly touch on the implications for human evolution given our species' evolutionary history marked by frequent expansion and adaptation to novel environments. We conclude by proposing several experiments and models with testable predictions following from the founder sociality hypothesis.

## Graphical abstract

We propose the founder sociality hypothesis to describe a novel evolutionary force shaping social species' behavioral ecology. After developing three specific predictions of social dynamics arising as species expand into new environments, we review three species predicted to have undergone such evolution and one species of baboons evidencing long‐lasting change in sociality from sudden shifts in dynamics. Finally, we form testable, concrete hypotheses to further study social founder effects.

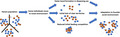

## 1 INTRODUCTION

### 1.1 Background

The extended evolutionary synthesis has called for additional perspectives in evolutionary theory beyond the gradualism through individual mutations emphasized in the modern synthesis (Laland et al., 2015; Pigliucci, 2007). Niche construction theory has gained attention since the 1990s as a mechanism of inheritance beyond culture and genetics, where researchers have proposed that modification of ecological environment inherited by the next generation is a key factor in evolution (Laland et al., 1999; Odling‐Smee et al., 1996). Social niche construction has expanded the reach of these proposals to the social environment as well (Saltz et al., 2016). Punctuated equilibrium emphasizes periods of relative stability and rapid in change in species, as opposed to constant, slow change, sometimes called phyletic gradualism (Gould & Eldredge, 1993). Gene‐culture coevolution presents another case of nongenetic inheritance, focused on how differences in behavior can lead to genetic evolution, shaping population structure and diversity in both human (Gintis, 2011) and nonhuman (Whitehead et al., 2019) animals. In this paper, we propose the founder sociality hypothesis, which suggests that the social dynamics arising from expansion into novel and underexploited habitats can lead to altered and persisting social evolutionary changes in the founder compared with parent populations.

Within the modern synthesis, founder effects describe changes in genotype and phenotype between a novel population expanding to a new environment and its parent population due to differential distribution of traits in the founder population (Mayr, 1942). First described by Ernst Mayr, founder effects are a special case of genetic drift and make up a part of the modern synthesis alongside mutation (random novel genetic changes), gene flow (transfer of genes between populations), and natural selection (differential reproductive success) (Huxley, 1942; Mayr, 1942). While social dynamics are a kind phenotypic variation which are subject to founder effects, few theories have explicitly addressed the additional possibility of altered social dynamics directly deriving from the social environment of small founder populations in novel habitats.

The founder sociality hypothesis suggests that as species expand into newly available and underexploited habitats, there will be differential survival and reproduction of individuals within founder populations based on their ability to form bonds and mate with other founders, and survive in an environment which initially has a low population and thus low competition. It further suggests that when these traits can be passed down either genetically or socially, it can lead to lasting differences in sociality between the parent and founder populations as future generations adapt to this social environment. Individuals expanding to new environments may be selected not only by the ecology of the new environment but by the altered social dynamics of the founder populations. Niche construction theory can be applied to the social environment as well as the ecological environment, where generations inherit social structure and social organization from the previous generation in addition to local ecology and genes. The founder sociality hypothesis highlights migration and habitat expansion into previously unexploited niches as an important instantiation of punctuated change and an important social evolutionary force. The founder sociality hypothesis may additionally be tied to gene‐culture coevolution. As new populations' expansion leads to population‐level differences in social behavior, these altered social dynamics are inherited and persist across generations, leading to genetic selection as a result of this novel social dynamic. The founder sociality hypothesis may be a key dynamic in the species' early selection and may have lasting effects on the novel populations' social structure.

After explaining our theoretical motivation, we review a motivating case of immediate but lasting changes in social dynamics in a population of olive baboons, with sustained influence on social behavior and hormone profiles across generations. In the next section, we summarize three nonhuman species or populations which have expanded into novel environments and show remarkably similar social differences from their parent population predicted by the founder sociality hypothesis. We then discuss the possibility of applying theory to humans, discuss the relation between the founder sociality hypothesis and other evolutionary hypotheses such as self‐domestication, and finally propose several direct tests of these predictions in humans, extant species, and evolutionary models.

### 1.2 Theoretical motivation for the founder sociality hypothesis

Before reviewing candidate species for the founder sociality hypothesis, we explain theoretical motivation for the proposal. Initial populations expanding into novel environments may experience a significantly different social environment in establishing groups and finding reproductive success which can result in long‐term changes in social structure to which subsequent generations adapt. This novel social environment, rather than differing ecological conditions alone, may lead to a suite of behavioral changes. In this paper, we focus on the more specific cases where a parent population expands into a newly available niche with initial lack of competition and which is separated in some way from the parent population. For example, habitat expansion into an environment for which there was a previous barrier that suddenly became available is a case where the founder sociality hypothesis comes into play.

In the remainder of this paper, we focus especially on species that form territorial, mixed‐sex groups. We do this in order to form more specific predictions about how the theoretical motivation for the founder sociality hypothesis has influenced their evolution and especially the similarity between the species reviewed in the next section. While we predict the founder sociality hypothesis can have wider ranging applications to species with diverse social structures, we here focus on species with more specific socio‐ecologies in order to more clearly and precisely suggest changes that may arise from differences in founder sociality. In such species with territorial, mixed‐sex groups, we propose three predictions on the change in sociality due to expansion into new habitats: relatively increased strength of social bonds between nonkin of the dispersing sex alongside relatively decreased strength of social bonds between the nondispersing sex, reduced territoriality, and increased interindividual tolerance.

Regarding the first prediction, in species with sex‐biased dispersal, the sex which does not disperse almost never forms social bonds with strangers and is often aggressive toward unfamiliar individuals of the same sex. The dispersing sex, however, must join existing social groups made up nearly entirely of strangers and form tolerant social bonds. If individuals expand into novel environments without existing groups, the dispersing sex, which has evolved to be able to form tolerant social bonds with strangers upon emigrating, could thus be predicted to more rapidly form novel social bonds. The nondispersing sex, with less ability to form bonds with strangers, may have difficulty initiating tolerant interactions with unfamiliar individuals, while the dispersing sex would be more able to form such associations. In the initial context, we suggest not absolutely stronger bonds between the dispersing sex than in the parent population, but relatively stronger bonds than the nondispersing sex in forming the core of a novel social group. The nondispersing sex in this environment would need to find and be accepted by the newly forming groups in order to achieve reproductive success and those more able to form such tolerant associations would be more successful. As bonds overall strengthen in the founder population, this relative shift of bond strength in both sexes can remain, shifting the social dynamics of the group. Through subsequent generations, due to the initial change in relative bond strength in the dispersing and nondispersing sex, the pattern can continue as groups become better defined and more cohesive. Over time, this can lead to a stable bond structure involving stronger bonds in the dispersing sex compared with the nondispersing sex. While we do not suggest purely that migrants must be lone individuals, we predict similar effects where some individuals may have expanded into the new environment together. In such cases, whether the nondispersing sex comes as lone individuals or small clusters, they will initially still have difficulty integrating and forming tolerant bonds with other individuals as novel groups are established without clear territories. The dispersing sex will not be subject to these challenges due to their ability to join groups and establish bonds with unfamiliar individuals. In the initial abundance of resources larger group sizes may be supported, which may cause these differences to become especially pronounced. This results in the founder population having stronger bonds in the dispersing sex as the subsequent generations adapt to this social environment.

In addition, upon entering the new habitat territories would be undefined, and with abundant food, there would be a significantly lower fitness benefit in defending a clearly defined territory. Without the fitness benefit from territorial defense of limited resources and without a clearly defined, closely related ingroup, engaging in outgroup aggression would likely result in high fitness costs and could be selected against in the founder population. Similarly, in an initial context of relative ecological richness, there is less to gain from direct aggression. There would likely be fewer circumstances of competition over limited resources upon entering the new habitat, and aggressive individuals may experience a loss of fitness from engaging in aggression with conspecifics during cofeeding. Tolerant individuals instead may have higher success by avoiding such costly aggressive interactions.

**FIGURE 1:**
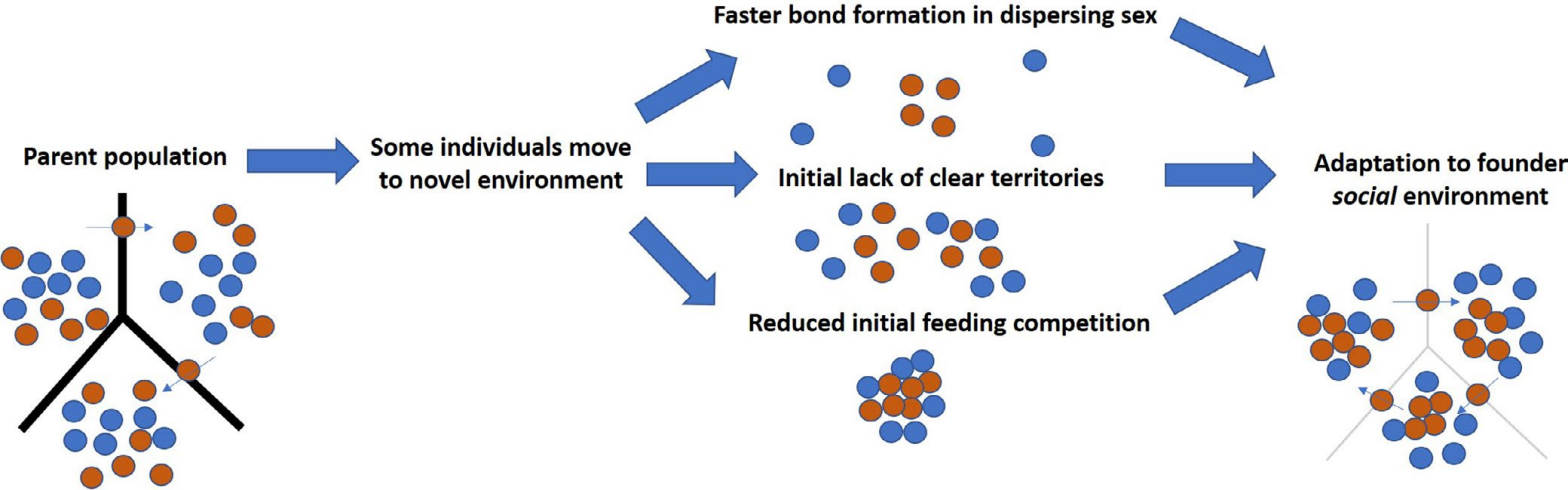
Illustration of founder sociality hypothesis

These predictions apply to the founders upon entering and forming initial groups in the novel environment. Although these pressures may dissipate over generations as consistent groups stabilize, territories become more clearly defined, and the initial abundance of resources is depleted, we predict that the initial and radically different social environment can lead to lasting changes in the species' sociality, especially when there is dysconnectivity (whether physical, social, or environmental) between the parent and founder populations. Given such changes in the founders, subsequent generations will be born into and must adapt to the altered social environment alongside the ecological environment. Individuals born into this environment will have differential fitness based on their ability to integrate into the existing social dynamics of the group. As the social structure stabilizes, these altered dynamics may yield lasting and significant changes to the population's sociality (Figure 1).

### 1.3 A motivating example of durable changes in social structure from initial change in olive baboons

A major question for the feasibility of the founder sociality hypothesis is the lasting effect of varied social structure, as opposed to the population returning to an equilibrium once individuals and groups have formed a stable population. Evidence for the durability of sudden changes in social dynamics comes from a troop of olive baboons (*Papio anubis*) in Kenya.

In the mid‐1980s, a tuberculosis outbreak killed half of the males of one group (Sapolsky & Share, 2004). Importantly, aggressive males disproportionately were killed (the aggressive males occasionally fed on garbage which became infected, killing all those who ate the garbage), leaving only those with relatively low aggression (Sapolsky & Share, 2004). Persistent changes in rates of aggression and hormone levels were observed a decade later. Most significantly, these changes persisted despite none of the original males remaining in the group and being fully replaced by immigrants from other groups, indicating that the immigrant males adapted to the social environment of the relatively unaggressive troop (Sapolsky & Share, 2004). As the baboons are female‐philopatric, each of the males in this subsequent study period would have been born into a more typical baboon social environment. Remarkably, they adapted their behavior to the social environment of the group to which they immigrated. This suggests that this sudden change in social dynamics, such that the group contained primarily nonaggressive males, was sufficient to produce generational change in the social behavior of the group. Immigrant males adapted to the social environment of the relatively unaggressive troop (Sapolsky & Share, 2004).

It is feasible to suggest that such a sudden change could occur through the initial success in survival and mating of founder populations. In particular, the ability to form social bonds with strangers, reduce territorial aggression, and tolerate cofeeding with conspecifics could have had a sudden, strong impact on fitness of founder populations and persist through generations, ultimately shaping the social environment under which future generations evolved. In the case of the population of baboons reviewed here, change in ecology is not the sole factor which shaped the group dynamics and thus the social environment to which immigrants and infants must adapt, but also the sudden reduction in aggression of ingroup males. While the immigrant males adapted their behavior through plasticity as they arrived in this new social environment, among the females permanently residing in this group there may be differential fitness to this particular social environment. Similarly, while we emphasize plasticity in social behavior in the founder population forming the novel social dynamics we predict, subsequent generations can experience selection for their fitness in such an altered social environment.

## 2 REVIEW OF SPECIES

### 2.1 Bonobos (*Pan paniscus*)

Bonobos may be one of the most direct applications of the founder sociality hypothesis. Despite diverging <2 million years ago and having similar foraging ecologies, the social differences between chimpanzees and bonobos are pronounced (Hare et al., 2012; Hare & Yamamoto, 2017). Bonobos, compared with chimpanzees, are characterized by strong social bonds between females, affiliative intergroup relations, and relatively tolerant social structure (Hare & Yamamoto, 2017). Evolving on opposite banks of the Congo River, recent geological work has shown that the Congo River is older than had been assumed and that bonobos are likely descendants of an initial population of a common ancestor who crossed the river at a low point (Takemoto et al., 2015). The founder sociality hypothesis proposes that initial individuals who crossed the Congo River experienced strong initial selection for their ability to form new groups and best exploit then‐underexploited resources available and that subsequent generations evolved under this novel social environment. Under this hypothesis, a more chimpanzee‐like common ancestor in some important respects is assumed (following Hare et al., 2012 and Wrangham & Pilbeam, 2002), though we recognize this is an oversimplification.

#### 2.1.1 Social bonds in dispersing sex

In both chimpanzees and bonobos, and thus likely the common ancestor, females disperse at sexual maturity, whereas males stay in their natal groups (Gerloff et al., 1999; Hare & Yamamoto, 2017; Ishizuka et al., 2020; Stumpf et al., 2018; Thompson, 2013). Male chimpanzees form alliances that can last for decades, while females typically forage in smaller subgroups or alone (Chapman & Wrangham, 1993; Hayaki et al., 1989; Nishida, 1983; Williams et al., 2002; Wrangham, 2000; Wrangham & Smuts, 1980). In bonobos, on the other hand, females have closer bonds and typically form the core of the group with males more peripheral (Furuichi, 2011). While chimpanzee communities have strict hierarchies with all adult males higher ranked than all females (Hayaki et al., 1989; Luef & Pika, 2019; Muller & Wrangham, 2004; Riss & Goodall, 1977), bonobo dominance is focused instead on a core group of adult females, with relatively weak bonds between males (Furuichi, 1997, 2011; Surbeck, Boesch, et al., 2017; Surbeck, Girard‐buttoz, et al., 2017; Tokuyama & Furuichi, 2017).

If individuals crossed the Congo River, females could be predicted to more rapidly form novel social bonds. Male chimpanzees do not form social bonds with strangers of the same sex in the wild and engage in lethal intergroup aggression (Wilson & Wrangham, 2003). A chimpanzee‐like common ancestor would likely thus find difficulty associating with unfamiliar males. Female chimpanzees and bonobos, however, must join existing social groups made up nearly entirely of strangers and form tolerant social bonds. Females may have been able to form bonds with strangers much faster than males upon crossing the Congo River. In order to reproduce, males would need to find females and be accepted by the newly formed female groups. Male offspring, then, may find their best chance of mating through proximity with their mother, who may be tolerant of other females. This hypothetical social dynamic is very similar to what is actually observed in wild bonobos, where a core of closely bonded females are central to the group, and more peripheral males seek mating opportunities through being accepted by the core female coalition, and males have significantly higher mating success if their mother is in the group (Furuichi, 1997, 2011; Surbeck et al., 2011).

#### 2.1.2 Territoriality

Chimpanzees are characterized by aggressive and competitive intergroup relations (Wilson & Wrangham, 2003). Their territories are well defined, and coalitions will engage in border patrols where several individuals of one community will range at the border of their territory, seemingly in search of lone outgroup individuals, which are often aggressive and sometimes lethal (Wilson & Wrangham, 2003). Bonobo communities, on the other hand, often contain areas of overlap, and groups will often play, groom, and forage together during intergroup encounters (Hohmann, 2001; Itani, 1990). Captive juvenile bonobos will choose to share food with strangers given the choice, and even prefer unfamiliar to familiar individuals (Tan & Hare, 2013). Bonobos clearly differ strongly from chimpanzees in their form of intergroup relations and we propose the founder sociality hypothesis may be, at least in part, responsible for these differences. Upon crossing the Congo River without clear territories, more tolerant intergroup associations may have emerged which were maintained through subsequent generations.

#### 2.1.3 Tolerance

Bonobos are also characterized as more tolerant than chimpanzees. In cofeeding observations, bonobos have been described as more tolerant while exhibiting higher frequency of play (Enomoto, 1990; Goldstone et al., 2016; Hare et al., 2007; Kano, 1980; Kuroda, 1984; Nurmi et al., 2018; Palagi & Cordoni, 2012; Parish, 1994; Yamamoto, 2015; Yamamoto & Furuichi, 2017). In captive cooperation experiments, bonobos cooperate more successfully than chimpanzees, especially when food can be monopolized (Hare et al., 2007). Dominant chimpanzees often attempt to monopolize food, decreasing motivation of subordinates to cooperate (Hare et al., 2007). When paired in dyads with higher tolerance, chimpanzees cooperation was more similar to that of bonobos, suggesting tolerance is the driving force in the species differences (Hare et al., 2007; Melis et al., 2006). Within‐group aggression differs between the species, with significantly lower intensity of aggression in bonobos compared with chimpanzees (Hare & Yamamoto, 2017) and lower rates of male within group aggression in bonobos (Surbeck, Boesch, et al., 2017). Chimpanzees will engage in lethal aggression even toward members of their group, while bonobos in the wild have never been observed to kill a conspecific (Hare & Yamamoto, 2017; Pruetz et al., 2017). We suggest these differences may be explained in part by the founder sociality hypothesis of early bonobo ancestors upon crossing the Congo River.

#### 2.1.4 Discussion

Major theories about the selection pressures responsible for bonobos' tolerant social structure with strong female bonds include a relatively rich environment of evolutionary selection, potentially due to lack of feeding competition with gorillas for terrestrial vegetation (Hare et al., 2012; Wrangham, 1993), and/or an extended estrous and pseudoestrous period reducing conflict between males for access to estrous females (Furuichi, 2011; Hare et al., 2012). These proposals can account for much of the variation observed between bonobos and chimpanzees, but typically are argued through analogy of stable chimpanzee‐like common ancestors living in slightly different ecologies. Ecological evidence on nutritional availability has been mixed, leading many authors to conclude that food availability in their habitats alone cannot fully account for the variation in foraging patterns observed between the two species (Furuichi, 2020, Furuichi, 2009; Hohmann et al., 2009; Yamakoshi, 2004; but see Malenky & Wrangham, 1994). Under the founder sociality hypothesis, the lack of competition with gorillas or greater fruit or herb volume in bonobos environments, for which the evidence is unclear (Furuichi, 2009, 2020; Hohmann et al., 2009; Yamakoshi, 2004), is not necessary. Comparisons between chimpanzees and bonobos in areas with varying ecologies are worthy of direct investigation, as bonobos' range has been shown to include environments such as forest–savanna mosaic (e.g., Nkala forest; Onishi et al., 2020), where traditional ecological theories would predict more chimpanzee‐like social dynamics. Furuichi (2011) similarly suggests a population bottleneck upon crossing the Congo River as forming a major part of bonobos' evolutionary history and emphasizes that small genetic changes causing, for example, an extended female estrus, can lead to development of entirely different social system. We specify here in addition that the initial differences in the founding population may be due especially to the social needs of establishing novel populations through bonding with strangers, in then underexploited niches without clear territories and that genetic adaptations to this social environment could follow. Under this hypothesis, upon crossing the Congo River in an initial resource abundance without defined territories females would initially form associations to which males attempt to join for reproductive opportunities. Offspring born into this environment would then experience different selection pressures on sociality than the parent population and lead to stronger female bonds, weaker male bonds, increased tolerance, and reduced territoriality even as the niche is occupied by the expanding population.

Although it is not possible to test these hypotheses in the wild, the social dynamic predicted by several individual chimpanzees crossing the Congo River and adapting to the novel rich environment populated largely by strangers maps neatly onto the observed social dynamic in the wild.

### 2.2 Domestic dogs (*Canis familiaris*)

Domestic dog behavior may also benefit from appeals to the founder sociality hypothesis. Recent proposals of dog evolution have emphasized early natural selection rather than artificial selection by humans (Coppinger & Coppinger, 2001; Hare & Woods, 2013). These self‐domestication proposals hypothesize that early wolves started to take advantage of the as yet unexploited ecological niche of feeding on prey animals in human settlements as well as human garbage (Coppinger & Coppinger, 2001; Hare & Woods, 2013). In such proposals, early wolves experienced selection against aggression and reactivity in order to best exploit the niche without suffering aggression from humans (Coppinger & Coppinger, 2001; Hare & Woods, 2013). The founder sociality hypothesis additionally adds that self‐domestication did not occur purely through selection on groups or individual wolves in human modified environments, but that the social behaviors enabling successful colonization and mating were key drivers of the early stages of dog self‐domestication.

#### 2.2.1 Social bonds between dispersing sex

In wolves, both sexes disperse at sexual maturity (Cassidy et al., 2017; Mech, 1987; VonHoldt et al., 2010). Wild wolves of either sex are rarely observed with affiliative bonds between same‐sex nonkin (Boitani & Ciucci, 1995; Cassidy et al., 2017; Mech & Boitani, 2003). Further, individuals of both sexes engage in outgroup aggression, including lethal aggression (Cassidy et al., 2017; Mech, 1994, 2003). Because both sexes disperse in wolves, we do not predict a shift in the bond strength of males compared with females as the first individuals expand. However, we predict that in this initial environment, without clear territories and where interindividual tolerance is higher, that nonkin of both sexes will begin to form tolerant social bonds. Similar to the other species, as groups gradually become established, social bond strength will increase. Unlike parent populations of wolves, where same‐sex nonkin rarely associate, in the initial setting with more abundant resources same‐sex nonkin will have greater fitness by tolerating one another's presence, which can lead to formation of bonds as groups become established. The founder sociality hypothesis thus predicts increased strengths of social bonds between both male and female nonkin. Domestic dogs, in support of this hypothesis, frequently form bonds with unfamiliar same‐sex conspecifics (Boitani et al., 1995; Daniels & Bekoff, 1989; Pal et al., 1999). Even in free‐ranging settings, groups typically contain multiple breeding individuals of the same sex (Boitani & Ciucci, 1995; Boitani et al., 1995; Daniels, 1983a). Although genetic studies are lacking, the group structure of free‐ranging dogs, compared with wolves, is characterized by nonkin group affiliations of both sexes (Boitani & Ciucci, 1995).

#### 2.2.2 Territoriality

Territoriality is also significantly reduced in dogs compared with wolves. Free‐ranging dog social dynamics vary widely across studies, but rarely include the kind of clearly defined, mutually exclusive territories common in wild wolves (Daniels, 1983b; Daniels & Bekoff, 1989). Wolves in Denali, for instance, experience mortality rates due to intraspecific aggression as high as 39%–65% (Mech, 2003). In dogs, however, there is little evidence for strict territoriality. In some study sites, authors have concluded there is no evidence for territoriality, and at others, home ranges are described but involve significant overlap and very few instances of outgroup contact aggression (Boitani & Ciucci, 1995; Daniels, 1983b).

#### 2.2.3 Tolerance

Although the characterization has been challenged in recent years, dogs are often described as significantly more tolerant than wolves (Hare et al., 2012; Hare & Tomasello, 2005; Hare & Woods, 2013; but see Range et al., 2015). Tolerance has been proposed as a driving force of dog evolution that enabled the human cooperation seen in modern dogs (Hare & Woods, 2013). Though dogs and wolves both can form hierarchies, especially in captivity, characterizations of both have been challenged and updated (e.g., Mech, 1999). Of note, however, is that studies such as Bradshaw et al. (2009) failed to find evidence for an overall hierarchy among free‐ranging dogs, Pal et al. (1998) did not observe ritualized dominance and submission signals more often seen in wolf packs, and Pal et al. (1999) observed extremely low rates of female–female aggression which would be expected if there were dominance competition for reproductive success. Bradshaw et al. (2009) review the use of dominance in reference to dog compared with wolf hierarchies and behavior and generally conclude there is little evidence for wolf‐like dominance structures. Future work should focus more on comparing the two with the same measures, and the best way to characterize dog dominance warrants more empirical work, but in any case dominance behaviors in dogs and wolves appear to differ strongly. In addition, there are no reported cases of infanticide in dogs and little evidence for reproductive suppression which occur more frequently in wolves (Boitani & Ciucci, 1995; Macdonald & Carr, 1995; Pal et al., 1999). More direct tests are needed, but dog tolerance is itself often emphasized as a key difference between the two species (Hare et al., 2012; Hare & Woods, 2013).

#### 2.2.4 Discussion

Although proposals of dog self‐domestication invoking adaptation to the novel niche predict strong founder effects, especially in their human‐directed behavior, no paper has explicitly acknowledged the changes in conspecific social behavior predicted by expansion into a new niche. This view is in contrast to the view that groups of wolves expanded together into human dominated environments and experienced gradual selection on reactivity toward humans, eventually becoming tolerant of unfamiliar conspecifics. The founder sociality hypothesis predicts that initial exploration of the novel niche available to wolves in human settlements led to immediate changes in social dynamics as individuals needed to form affiliations and mate with unfamiliar individuals, were largely unable to maintain clear territories (leading to lower motivation for territoriality), and experienced lower levels of direct competition with one another for resources and that this altered initial social dynamic was inherited by the next generations.

### 2.3 Zanzibar red colobus (*Piliocolobus kirkii*)

Bonobos and dogs are strong candidates for species that may have experienced the founder sociality hypothesis, but neither species provides an opportunity to directly study the process as it happens. Zanzibar red colobus monkeys are an interesting case where two adjacent populations exhibit remarkable differences in social structure. Adjacent to Jozani Chwaka Bay National Park, a relatively natural habitat, there are numerous spice farms that Zanzibar red colobus monkeys have colonized (Siex, 2003). Although a lack of studies limits knowledge of gene flow and connections between these populations, Siex (2003) did not observe any migrations between the populations (though did not observe any marked individuals in the forest population disperse). In any case, the habitats are distinct and the farm populations subject to the founder sociality hypothesis upon the first expansion, likely shaping the early group social dynamics and subsequent social environment even if there remains some gene flow between the populations.

#### 2.3.1 Social bonds of dispersing sex

In most populations of red colobus monkeys across Africa, females are the primary dispersers (Struhsaker, 2010), and thus, the founder sociality hypothesis predicts a relative increase in the strength of female social bonds and relative decrease in the strength of male social bonds. Compared with the groups living in Jozani Chwaka Bay National Park, females in the farm‐living groups groom one another significantly more (Siex, 2003). In contrast, no adult males in the farm‐living groups were ever observed to groom one another in a detailed study by Siex (2003), a marked difference from any other species or population of red colobus where comparable studies have been performed (Struhsaker, 2010). In addition, the farm population are the only population of any red colobus monkey where female–female stylized presents have been observed (Struhsaker, 2010). Siex (2003) suggests there may be stronger female–female bonds among farm‐living Zanzibar red colobus than any other taxa, while males are characterized by relatively weak bonds compared with other taxa.

#### 2.3.2 Territoriality

The farm population also differs from those in Jozani Chwaka Bay National Park in having significantly higher home range overlap (Siex, 2003). In addition, adult males transfer between groups at a higher rate than any other taxa of red colobus (Siex, 2003). Both males and females transfer between groups, including adults and juveniles (Siex, 2003). No resident female aggression toward immigrants has been observed (Struhsaker, 2010). These findings suggest territoriality may be reduced in the farm population of Zanzibar red colobus, as predicted by the founder sociality hypothesis.

#### 2.3.3 Tolerance

Within groups in both populations, the farm groups were found to be more cohesive and spent significantly more time in close proximity to one another (Siex, 2003). They additionally spend more time in both play and grooming than the forest population (Siex, 2003). Zanzibar red colobus in the farm populations spend twice as much time engaged in social activities than other species of red colobus monkey (Siex, 2003). In fact, Zanzibar red colobus living in farm habitats live at the highest density of any nonhuman anthropoid (Struhsaker, 2010). These findings are consistent with the prediction of increased tolerance in groups arising from the founder sociality hypothesis. Although rates of aggression are relatively high in the spice farms, even compared with the groups within the boundaries of the National Park, this may be caused by the relative frequency of cofeeding in close proximity alongside the overall higher density (Siex, 2003). More data are needed to understand the exact causes of aggression in this population, though it is suggested to be caused by more recent population compression (Siex, 2003).

#### 2.3.4 Discussion

**FIGURE 2:**
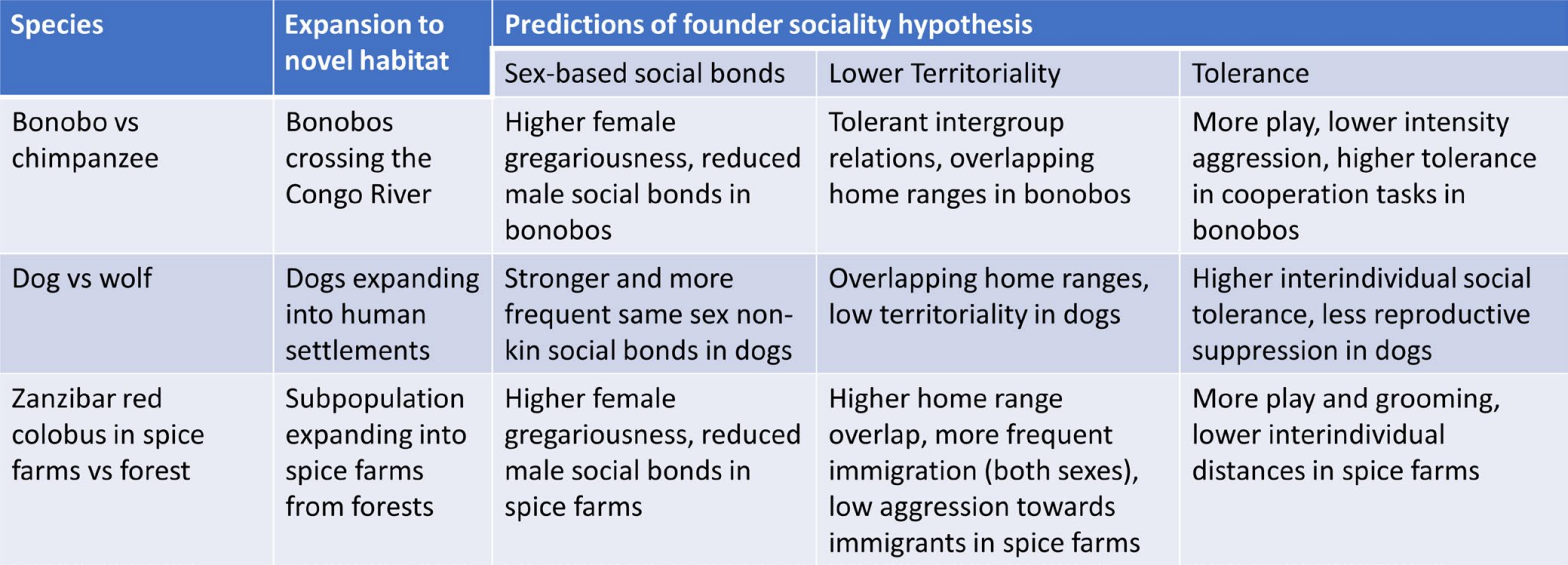
Summary of predictions and findings in the species in Section 2

Consistent with the predictions of the founder sociality hypothesis, Zanzibar red colobus monkeys living in spice farms have relatively strong female social bonds and relatively weak male social bonds, increased home range overlap, and increased grooming and play compared with the forest populations and other red colobus taxa. Although the populations have been separated for a short time (at the earliest since the farms were established following the 1964 revolution), the social structure has changed drastically. Importantly, this cannot be explained purely by increased feeding resources, as per capita feeding opportunity is lower in the farm population (Siex, 2003). This contrasts with bonobos, where the same changes are predicted to be in part due to reduced feeding competition (Hare et al., 2012). The founder sociality hypothesis suggests that these social changes were a direct result of the altered social dynamics of the founder population who colonized the spice farms, where initial competition was low and females were better able to form novel social groups in undefined territories while males struggled, and that offspring in the farm populations then experienced selection to this novel social environment in addition to the novel ecological environment (Figure 2).

## 3 DISCUSSION

### 3.1 General discussion

If the founder sociality hypothesis has significantly shaped the evolution of species and populations expanding toward new environments, such as bonobos, domestic dogs, and Zanzibar red colobus monkeys, it is natural to ask whether humans may have experienced similar selection. Humans of both sexes in both agricultural societies and hunter gatherers are able to form lifelong affiliative bonds with nonkin (Apicella et al., 2012; Hill et al., 2011, 2014; Tomasello et al., 2012), live in overlapping territories with frequent migration and cross‐cultural interaction (Hamilton et al., 2007; Hill et al., 2011; Layton et al., 2012; Robinson & Barker, 2017), and show rates of aggression between 2 and 3 orders of magnitude less frequently than chimpanzees (Wrangham, 2018; Wrangham et al., 2006). Humans are thought to have experienced several periods of habitat expansion and modification since divergence from our common ancestor and have colonized ecological niches in many different environments (Kendal et al., 2011; Templeton, 2002; Tomasello et al., 2012; Vrba et al., 1995), so it is feasible to suggest that there were several instances where the effects predicted by the founder sociality hypothesis played an important role in the evolution of our species' behavior and social dynamics. Interactions with strangers, the frequency of nonkin and cross‐group social bonds of both hunter gatherers and agriculturalists, and humans' flexibility in social group formation may be in part a result of our species' frequent habitat expansion. In this paper, we merely suggest the human case as an interesting avenue of future study given the frequent expansion in human evolutionary history and consistency with the predictions of the hypothesis when compared with chimpanzees. We additionally emphasize that these traits can be found across human groups and are most consistent with the predictions of early *homo* expanding to habitats and niches not occupied by any other hominins, as opposed to more recent expansions. Future work should conduct explicit tests about the possibility of the founder sociality hypothesis in humans and how to differentiate them from other proposals on human evolution.

Another element of the founder sociality hypothesis that should be further explored is whether each of the three factors identified here are selected in founder populations, or if they represent a more general tendency against reactive aggression, as in the self‐domestication hypothesis. In fact, all three species reviewed here, as well as humans, have been proposed as candidates for self‐domestication, and the self‐domestication hypothesis is suggested as central to convergence of behavior between the species reviewed here (Hare, 2017; Wrangham, 2019). The self‐domestication hypothesis is largely consistent with the founder sociality hypothesis. While the founder sociality hypothesis is predicted in shaping the initial effects responsible for altered social dynamics, self‐domestication can be understood as the evolutionary pathway by which the changes occur, in particular the force of selection acting on subsequent generations in the novel social dynamic. A key difference, however, is the self‐domestication proposes the behaviors to evolve together under selection against reactive aggression, while the founder sociality hypothesis predicts each is specifically favored for their role in the establishment of early populations. To piece out the factors, comparisons of closely related species and the same species across different habitats should be conducted to examine whether factors reliably covary or can be altered independently. The decrease in bond strength among individuals of nondispersing sex cannot easily be explained by reduced reactive aggression alone. Additionally, as early as Darwin as well as ongoing research today suggests coevolution of ingroup cooperation with outgroup aggression (Bowles, 2009; Choi & Bowles, 2007; Darwin, 1871; Yamamoto, 2020) and preliminary tests in chimpanzees have found evidence for a correlation in both wild (Samuni et al., 2019) and captive (Brooks, Onishi, et al., 2021) contexts. Whether the three predictions presented in this paper are independent or stem from a common evolutionary pressure will be an important piece of evidence in testing the validity of the founder sociality hypothesis.

As mentioned in the introduction, how the general hypothesis developed here applies to species with more varied social systems should be studied explicitly. While we here focused on species which form territorial, mixed‐sex groups in order to ground specific empirical predictions, we do not believe the general phenomenon of lasting social changes as a result of habitat expansion to be limited to such species. Future work should form specific hypotheses about how the novel socio‐ecological dynamics in establishing founder populations would act on species with different grouping styles, for example harem groups, species with more flexible grouping dynamics, and species without clear territories. This line of investigation can further clarify general and specific elements of the founder sociality hypothesis across species.

### 3.2 Future directions

Although the founder sociality hypothesis can explain many traits observed in bonobos, dogs, Zanzibar red colobus monkeys, and possibly some in humans, direct evidence and tests of its predictions are required. It is more difficult to test these species directly, especially regarding the initial changes, as the hypothesis proposes the effects occurred immediately upon expansion to their current environments. However, other species are now expanding into new habitats which may provide clear tests of the hypotheses. We predict the founder sociality hypothesis to be most important in habitat expansions toward a separated and underexploited novel niche, in contrast to gradual expansion into bordering areas which over time become able to support the species' survival. We predict that species undergoing gradual habitat expansion in such a way will not demonstrate the pattern of sociality described here to the same extent. For instance, highly social invasive species are one example of species which are predicted to have experienced similar changes in social structure even in habitats of relative similarity. The difference in sociality between an invasive species in one part of its home range and a relatively similar ecology in an environment it has invaded is predicted to be larger than the difference between two patches of its native habitat with more pronounced ecological differences. Further, recovering and reintroduced species are reoccupying the same ecological niches they once filled, and thus can be predicted to experience effects of the founder sociality hypothesis, while no changes would be predicted by purely ecological models. Island populations provide another test of this hypothesis. For instance, the Awajishima and Shodoshima island populations of Japanese macaques show remarkable interindividual tolerance and may be good candidates to test predictions of the founder sociality hypothesis (Kaigaishi et al., 2019; Nakagawa, 2010; Zhang & Watanabe, 2007). Species who are thought to have arrived on islands from a mainland group are predicted to experience strong shifts in sociality from the founder sociality hypothesis, whereas populations inhabiting wider ranges that were split when islands became separated from the mainland, and thus already occupied the niche, are not predicted to display the same effects. Establishment of captive colonies of social species may also be suggested to be important to the founder sociality hypothesis, though the significant change in feeding ecology with constrained feeding times (thus lacking the same initial abundance of food) and more closed environment (producing more delineated territories) may differ too drastically from the situations proposed here for the same effects to emerge.

In addition, species recently expanding into urban environments, such as coyotes across North America, provide ideal candidate species. Coyotes historically lived only in the west coast of North America but have expanded across the entire continent and into towns and cities (Hody & Kays, 2018). At the same time, cities and towns have expanded into coyotes' original habitat (Hody & Kays, 2018). Coyotes adapting to urban areas in the east coast have been proposed as a candidate species to test predictions of the self‐domestication hypothesis and dog evolution (Brooks et al., 2020). The founder sociality hypothesis would predict that coyotes expanding eastward, particularly those expanding into underexploited ecological niches such as those provided by urban environments, would more directly be subject to the founder sociality hypothesis than either coyotes expanding eastwards into ecologies similar to their original niche through gradual expansion, or coyotes in areas where urbanization is encroaching on their native habitat. More specifically, the founder sociality hypothesis predicts that in human settlements in Eastern North America, coyotes will be more likely to develop social bonds with same‐sex nonkin, will show reduced territorial defense, and will display reduced conspecific aggression, compared with coyotes in both undisturbed environments and urban areas in the Western United States.

Human behavioral and psychological studies attempting to distinguish the founder sociality hypothesis from other proposed explanations of human's cooperative tendencies with strangers can provide further tests of the hypotheses. Minimal group paradigms may yield interesting results (see Diehl, 1990). For example, after assigning individuals into several minimal groups, performance on economic games in response to sudden recombination into mixed groups can be compared with performance of stable groups. Trust, competition, and tolerance could then be compared while controlling for other factors such as total resource availability, potential gains from stealing, and initial resource distribution. Outside of the laboratory environment, behavioral studies comparing immigrants, travelers, and expatriates to individuals in both their country of birth and country of residence could also prove insightful.

Hormonal studies may also be important tests. In the olive baboon case mentioned in the introduction, differential hormone profiles proved one of the clearest signs of persistent cultural shifts between groups. In addition, bonobos and chimpanzees have differing baseline levels of testosterone (Sannen et al., 2003) and differential ontogenetic patterns (Wobber et al., 2013), have differential changes in urinary hormones in anticipation of social competition (Wobber et al., 2010), and are affected differently by oxytocin (Brooks, Kano, et al., 2021). Dogs, compared with wolves, also have markedly different hormone profiles (Kikusui et al., 2019; Wirobski et al., 2021) and are similarly influenced differently by oxytocin (Nagasawa et al., 2015). The self‐domestication hypothesis likewise has emphasized hormonal shifts, especially the oxytocin system, as a key element in shifting reactivity and social behavior leading to the evolutionary shifts described (Hare, 2017; Herbeck & Gulevich, 2019). While hormonal studies of Zanzibar red colobus monkeys are missing, the hypothesis presented here suggests, compared with the forest groups, the farm population will show differences in baseline testosterone, cortisol, and will be affected differently by oxytocin. Hormone profiles may provide part of the physiological and endocrine basis by which the effects of the founder sociality hypothesis act and can be empirically measured and compared between populations and species.

Finally, theorists should build evolutionary models and develop hypotheses about how the founder sociality hypothesis relates to other proposed evolutionary forces, especially as part of an extended evolutionary synthesis. Parallel models between the ecological environment of niche construction theory and the social environment of the founder sociality hypothesis, especially in cases of punctuated equilibria through expansion to novel habitats, may yield relevant and important findings. Models which independently vary the three main predictions of the founder sociality hypothesis raised here can help distinguish the forces involved, and models simulating a novel population's survival and mating success in areas with undefined territories and social relationships can directly test the feasibility of early and lasting alterations to founder populations' social structure and the resulting selection pressures. Theoretical and empirical research should also explore how the founder sociality hypothesis fits within broader proposals for an extended evolutionary synthesis. Its precise characterizations and implications for social niche construction, gene‐culture coevolution, and punctuated equilibria should be a focus of ongoing work.

## 4 CONCLUSION

The founder sociality hypothesis may be important to the evolution of many species. Ecological theories are able to explain vast amounts of behavioral variation between closely related species but may underappreciate the role of altered social dynamics in founder populations. As evolutionary theorists increasingly focus on niche construction and dynamic as opposed to gradualist perspectives of evolution, the lasting influence of altered social dynamics in founders should not be neglected. Increased strength of social bonds between individuals of the dispersing sex, reduced territoriality, and increased social tolerance are three specific factors predicted to be directly selected by the founder sociality hypothesis in species that form territorial, mixed‐sex groups. Genetic bottlenecks, founder effects, and nongenetic inheritance have long been recognized as key drivers of natural selection, but the explanatory power of founder populations' social dynamics and the resulting social inheritance may as yet be underappreciated.

## CONFLICT OF INTEREST

The authors declare no conflict of interest.

## AUTHOR CONTRIBUTIONS

**James Brooks:** Conceptualization (lead); Writing‐original draft (lead). **Shinya Yamamoto:** Conceptualization (supporting); Supervision (lead); Writing‐review & editing (equal).

## Data Availability

No data are used in this paper.
